# Is the national health insurance scheme a pathway to sustained access to medicines in Nigeria?

**DOI:** 10.1186/s12913-024-10827-1

**Published:** 2024-03-29

**Authors:** Nkolika Uguru, Udochukwu Ogu, Chibuzo Uguru, Ogochukwu Ibe

**Affiliations:** 1 Department of Preventive Dentistry, Faculty of Dentistry, College of Medicine, University of Nigeria Enugu Campus, Enugu, Nigeria; 2 Health Science Centre, University of North Texas, Fort Worth, TX United States of America; 3 Health Policy Research Group, Department of Pharmaco-therapeutics, College of Medicine, University of Nigeria, Enugu Campus, Enugu, Nigeria; 4 Department of Oral and Maxillofacial Surgery, Faculty of Dentistry, College of Medicine, University of Nigeria Enugu Campus, Enugu, Nigeria

**Keywords:** Access to medicine, National health insurance scheme, Universal health coverage, Nigeria

## Abstract

**Objective:**

The debate surrounding access to medicines in Nigeria has become increasingly necessary due to the high cost of essential medicine drugs and the prevalence of counterfeit medicines in the country. The Nigerian government has proposed the implementation of the National Health Insurance Scheme (NHIS) to address these issues and guarantee universal access to essential medicines. Access was investigated using the 3 A’s (accessibility, affordability, and availability). This paper investigates whether the NHIS is a viable pathway to sustained access to medicines in Nigeria.

**Design:**

This was a cross-sectional study using a mixed-methods design. Both qualitative and quantitative methods were utilized for the study.

**Setting:**

This study was conducted at NHIS-accredited public and private facilities in Enugu State.

**Participants:**

296 randomly selected enrollees took part in the quantitative component, while, 6 participants were purposively selected for the qualitative component, where in-depth interviews (IDIs) were conducted face-to-face with NHIS desk officers in selected public and private health facilities.

**Results:**

The quantitative findings showed that 94.9% of respondents sought medical help. Our data shows that 78.4% of the respondents indicated that the scheme improved their access to care (accessibility, affordability, and availability). The qualitative results from the NHIS desk officers showed that respondents across all the socio-economic groups reported that the NHIS had marginally improved access to medicine over the years. It was also observed that most of the staff in NHIS-accredited facilities were not adequately trained on the scheme’s requirements and that most times, essential drugs were not readily available at the accredited facilities.

**Conclusion:**

The study findings revealed that although the NHIS has successfully expanded access to medicines, there remain several challenges to its effective implementation and sustainability. Additionally, the scheme’s coverage of essential medicines is could be improved even more, leading to reduced access to needed drugs for many Nigerians. A focus on the 3As for the scheme means that all facility categories (private and public) and their interests (where necessary) must be considered in further planning of the scheme to ensure that things work out well.

**Supplementary Information:**

The online version contains supplementary material available at 10.1186/s12913-024-10827-1.

## Introduction

One of the targets of the Sustainable Development Goals (SDGs) is linked to universal health coverage, which is one of the ways that governments can protect their populations from the financial hardship of out-of-pocket healthcare expenditures. Access to safe, effective, and quality essential medicines is central to achieving universal health coverage (UHC) and the improved health of the population. Health is, therefore the entry point for breaking the vicious cycle of poverty and under-development and transforming it into improved health status, sustainable development, and prosperity [1]. Countries around the world have adopted social health insurance programmes as a means to ensure access to health care while also, protecting patients from the financial risks of ill health [2, 3]. Thus, many African countries, including Nigeria, have established their own social health insurance [4].

The National Health Insurance Scheme (NHIS) was established by Decree 35 of 1999 to ensure that every Nigerian has equal access to good quality healthcare services [2]. The actual implementation of the NHIS commenced in 2002 and was consolidated in 2005 [2, 5]. The Formal Sector Social Health Insurance Program (FSSHIP) for employees in the formal sector, the Urban Health Self-employed Social Health Insurance Program (USSHIP), and the Rural Community Social Health Insurance Program (RCSHIP) are all components of the NHIS.

Access to medicines in health care systems has five dimensions: availability, accessibility, affordability, acceptability, and quality [6]. In a nutshell, access to medicines implies that individuals have access to the appropriate care and drugs at an affordable price and in a proper place [7]. Excerpts from other studies have shown that any health insurance programme directed or aimed at the public’s benefits should consider the three dimensions of access: availability, accessibility, and affordability [8]. In this instance, availability would mean adequate staff (skills mix), drugs, and equipment. According to the Global Health Workforce Alliance (GHWA), [9], availability is defined as an adequate supply and appropriate stock of health workers with the competency and skill set to meet the population’s health needs. Accessibility, according to GHWA [9] involves the equitable distribution of health workers by taking into account the demographic composition of the rural-urban mix and underdeveloped areas of the population. Affordability determines if a person or organization with limited resources can make a purchase or has sufficient income to pay for health care costs [6].

Despite the NHIS’s goal of providing affordable healthcare, access to medicine faces several challenges. Several challenges hinder access to medicine in Nigeria - inadequate funding, drug stockouts, inefficient supply chain management, and regional disparities in pharmaceutical infrastructure continue to impact access to medicine within the NHIS [10, 11]. To improve access to high-quality healthcare, more emphasis should be placed on access to the public sector. The focus should be on improving drug supply chains, ambulance services, clinical capacity at public clinics, and, most importantly, addressing the financial constraints faced by the socially disadvantaged. It is also imperative to think through how providers engage with patients in a way that strengthens their therapeutic alliance. Rekha, Wajid, Radhakrishnan, and Matthew [12] measured the accessibility index using a three-step floating catchment area in a geographical framework. Three variables were considered: the attractiveness of the health care centre, the travel time or distance between the service centre’s location and the residence, and the population demand for health care facilities. Another study [13] discovered that respondents who described quality in terms of the ease with which they received care or the short waiting time were 3.9 times more likely to use private facilities as their primary healthcare provider. The additional data collected indicated that the cheaper cost of service is 2.9 times more likely to predict the use of public health facilities than the usual health provider [13].

Essential drugs are seen as an essential component of UHC; they are an integral component of service delivery and meet the need for high-quality treatment. When establishing benefit packages, it is evident that considerable thought must be given to ensuring consistent access to quality-assured critical drugs [14]. National Essential Medications Lists (NEML), formularies, standard treatment recommendations, and efforts to provide access to cheap, quality-assured medicines are examples of policy instruments that contribute to the efficacy of the notion of essential medicines [14]. The low availability of medications in resource-constrained healthcare facilities is primarily connected to deficiencies in facility management, such as purchasing, distribution, or storage operations, as well as a lack of employee training and insufficient investment in medicines in general [15, 16]. As a result, guaranteeing fair and inexpensive access to drugs is a constant concern for healthcare systems [16].

NHIS’s main objective is to achieve equitable access to quality health care in Nigeria [17]. The key to achieving this is to focus on the 3As of access, namely, accessibility, affordability, and availability [18], without which healthcare outcomes in Nigeria will continue to be poor. However, despite the failures of the Nigerian healthcare system to improve the health of Nigerians, some studies suggest that if managed well, the NHIS could be a valuable tool for ensuring good healthcare delivery [19]. NHIS faced several inherent and systemic challenges in achieving universal health coverage for all Nigerians, which led to the signing of the new National Health Insurance Authority Act (NHIA). Although the NHIA addressed some of the shortcomings of the previous NHIS, several challenges still had to be addressed before it could be fully implemented, including a lack of financing for health and a shortage of healthcare workers etc. [20]. Even though the NHIA Act includes a well-thought-out strategy for pooling resources and improving risk pooling, these plans may not be feasible enough to mobilise this huge sum of money [20]. Furthermore, it has been shown that a significant hindrance to the country’s health insurance plan is a lack of funding [21–24].

Access to medicine is a critical aspect of healthcare, and the NHIS in Nigeria plays a pivotal role in shaping this access. The current study explores the dynamics of access to medicine within the framework of the NHIS, focusing on challenges and potential solutions to ensure equitable services for all beneficiaries. So, for the current study, the dimensions we will focus on are availability, accessibility, and affordability. By examining the 3As of access—accessibility, affordability, and availability—we can identify the gaps and barriers in the system and propose solutions for achieving equitable access to quality healthcare. This study can potentially inform policymakers and stakeholders on how to allocate resources better, improve infrastructure, and address the underlying issues that hinder healthcare delivery in Nigeria. In line with this, the objective of this study was to explore and emphasise the role access (availability, accessibility, and affordability) to medicine could play in the management and successful implementation of the NHIS towards achieving its universal health coverage goals. Specifically, we aim to assess the accessibility of healthcare services by examining factors such as geographic proximity, transportation infrastructure, and the availability of healthcare facilities. Additionally, we will examine the affordability of healthcare by analyzing the cost of services, health insurance coverage, and out-of-pocket expenses. Finally, we will evaluate the availability of healthcare providers and resources, including the distribution of healthcare professionals, medical equipment, and essential medications.

## Methods

This was a cross-sectional study which employed a mixed-method design. Both qualitative and quantitative methods were utilised for the study.

### Study setting

This study, which is part of a wider study, was conducted in NHIS-accredited public and private facilities in Enugu State in 2017. The state is situated in the southeast part of the country with a population of 4,411,100 million and an annual population growth rate of 3.0 [25]. The state has three senatorial zones (Enugu North, East, and West) with 17 local governments (3 urban and 14 rural). In addition, there are 962 health facilities, comprising 4 tertiary, 148 secondary (96 private and 52 public), and 774 primary (492 public and 282 private) health facilities. The federal government funds and operates three tertiary health facilities and one tertiary facility is operated and funded by the state government [26]. This paper focuses on the health facilities in the urban LGAs as they have the most facilities registered with the National Health Insurance Agency (NHIA) compared to those located in rural areas.

### Research instruments

The study used a questionnaire and an in-depth interview guide to collect data. The questionnaires were interviewer-administered and contained questions eliciting information on socio-demographic details, availability of needed medicines, affordability of needed medicines, sources of healthcare (public and private health facilities, pharmacy, patent medicine store etc.), distance between these health facilities and respondents’ household, perception of the quality of medicines, and patient satisfaction. The questionnaire has 60 questions and five sections - demographic information, illnesses and access to health services, opinions about obtaining medicines, experiences about medicines, assets and medicines expenditure. In the ‘Assets and Medicine Expenditure’ section (part five) of the questionnaire, an asset-based index consisting of information like ownership of a radio, bicycle, motor car, house, land, farmland, livestock and motorcycle together with the monthly household income was used to categorize the households into socio-economic status (SES) quartiles: least poor, poor, very poor and most poor. Also, to understand the level of access to medicines, respondents were asked questions about the kinds of illnesses they had and the level of access they had to medicines.

The IDI guide explored issues regarding access to medicines (accessibility, availability, and affordability), existing governance and medicine policy within the NHIS, supply of medicines (market forces), health information capacity, human resources, health financing, and service delivery.

### Data collection methods

A minimum sample size of 274 respondents was calculated for the quantitative component of the study using sample size calculation for the community survey. To account for non-responders, 10% was added, bringing the total number of respondents to 300. Respondents were drawn from the beneficiaries of the NHIS/ FSSHIP, which comprise civil servants in Enugu State. Three urban LGAs (Enugu East, North and Nsukka) were selected using purposive sampling by virtue of their location and because they have more NHIS-accredited facilities than the others. A sample frame of hospitals registered for NHIA was obtained from the above LGAs, and then 5 private hospitals and one public hospital were randomly selected from the frame using a simple random sampling technique. All the enrollees in the selected hospitals were beneficiaries of NHIS/FSSHIP. From the estimated sample size of 300 enrolees, 150 were randomly selected from the public facility, and 30 respondents were selected using consecutive purposive sampling from each of the five private facilities to give a total of 150 enrollees from private facilities.

For the qualitative component of the study, six face-to-face in-depth interviews (IDIs) (one from each of the facilities) ranging from 30 min to 1 h and 15 min with NHIS desk officers across the six healthcare facilities were conducted by two research assistants. The NHIS desk officers oversee the proper functioning of the scheme in the respective health facilities. All data collection tools were pretested before use in the study.

### Data analysis

Four questionnaires were not properly filled; those were discarded. Analysis of quantitative data was done using STATA 11. Frequency and percentages were computed and a test of association between dependent and independent variables. Chi-square tests were used to determine the level of treatment cost covered within the scheme. The tests of significance were carried out at a p-value ≤ 0.05. Data were presented in tables and narratives as in the result section. Principal components analysis (PCA) was used to generate the wealth index [27]. The SES index was disaggregated into quintiles - Q1 as the poorest, Q2 very poor, Q3 poor and Q4 as the least poor. The chi-square test was used to determine the SES differences of the key dependent variables.

For qualitative analysis, interviews were transcribed verbatim by the lead researcher, and then the lead researcher developed a codebook. Using NVivo 11 and the codebook, the main themes were identified from the transcripts, then colour-coded and analysed.

### Consent to participate

Those involved in the research gave verbal and signed consent.

## Results

### Socio-demographic characteristics

Of the 300 questionnaires distributed, 296 were filled correctly, giving a response rate of 98.6%. Females (67.6%) were more than males (32.4%). There were more respondents in the 31–40 years age category (33.8%). Response for those aged 0–12 years were given by parent(s). Majority of the respondents were Primary beneficiaries (68.2%). While their dependents (secondary beneficiary; their spouse, parent or child) made up 31.8% of respondents. About 72.6% of all study respondents were in paid employment, 9.8% were self-employed, and 6.1% were unemployed (see Table 1**)**.

**Table 1:** Respondents’ demographic characteristics (*N* = 296)

Variables	Frequency (%)
**Age**	
0–10	27 (9.1)
11–20	16 (5.4)
21–30	40 (13.5)
31–40	100 (33.8)
41–50	64 (21.6)
50 above	49 (16.6)
**Gender**	
Male	96 (32.4)
Female	200 (67.6)
**Primary Beneficiary**	
No	94 (31.8)
Yes	202 (68.2)
**Employment status**	
Unemployed	18 (6.1)
Self-employed	29 (9.8)
Paid employment	215 (72.6)
Not applicable	34 (11.5)

### Affordability of the scheme

The data in Table 2 suggests that the association between gender and level of coverage for treatment is not statistically significant (*p* ≤ 0.054). The study showed that 82.4% of respondents (male and female) indicated that their treatment costs were partly covered (See Table 2). Among those who indicated that treatment cost was partly covered, 72.9% were in paid employment, 11.5% were self-employed, and 4.5% were unemployed. Data on socioeconomic status showed that among the 82.4% that indicated that medicine costs were partly covered, 31.1% were in the poorest SES group; 25.4%, 21.1%, and 21.7% were in the least poor, poor, and very poor, respectively. About 82.8% of the respondents indicated that medicines for treating common medical conditions were affordable for those with low income (see Table 2).

**Table 2:** Level of Coverage for treatment cost (*N* = 296)

Variables	Level of coverage for treatment cost				Chi-square (p-value)
	Yes entirely (%)	Partly covered (%)		No (%)	
Male	8 (30.2)	85 (34.8)		3 (11.5)	**5.856 (0.054)**
Female	18 (69.8)	159 (65.2)		23 (88.5)	
**Total**	26 (100)	244 (100)		26 (100)	
**Employment status**					**9.748** ^**a**^ **(0.138)**
Unemployed	5 (19.2)	39 (16. 04)		8 (30.8)	
Self-employed	2 (7.7)	27 (11.1)		0	
Paid employment	19 (73.1)	178 (72.9)		18 (69.2)	
**Total**	**26 (100)**	**244 (100)**		**26 (100)**	
**Socio-economic status groups (SES)**					**9.782** ^**a**^ **(0.134)**
Poorest (Q1)	12 (46.2)	76 (31.2)	5 (19.2)	93 (31.4)	
Very Poor (Q2)	2 (7.7)	53 (21.7)	7 (26.9)	62 (20.9)	
Poor (Q3)	5 (19.2)	53 (21.7)	10 (38.5)	68 (23.0)	
Least Poor (Q4)	7 (26.9)	62 (25.4)	4 (15.4)	73 (24.7)	
**Total**	**26 (100)**	**244 (100)**	**26 (100)**	**296 (100)**	
**Drug affordability**					
**Affordability of drugs for common medical conditions for low–income earners**	**Frequency**			**Per cent**	
Yes	245			82.8	
No	21			7.1	
Don’t know	30			10.1	
Total	296			100.0	

Q1, 2, 3, 4– Quartile 1, 2, 3, 4

### Accessibility of the scheme

About 89.5% of respondents said they had acute health issues. The majority (94.9%) of respondents sought medical help, and 78.4% indicated that the scheme improved their access to care - availability, affordability, and accessibility. (See Table 3).

**Table 3:** Kind of illness and access to medicine (*N* = 296)

Kind of illness suffered	Freq. (%)
Acute illness (illness of short duration (days weeks)	265 (89.5)
Chronic illness (illness of long duration greater than 4 weeks)	31 (10.5)
**Responses on the number of those that seek for care on the illness above**	
Yes	281 (94.9)
No	15 (5.1)
**Did the scheme increase access to medicine?**	
Yes	232 (78.4)
No	55 (18.6)
Don’t know	9 (3.0)

### Availability of the medicines on the scheme

Our study showed that 78% of the respondents thought that the medicines on the scheme were of good quality (see Table 4). Also, 83.8% indicated the drugs are effective (see Table 4). These drugs were not always available. When asked, 47.6% of the respondents thought that they were not always available, while 45.6% of them believed that the drugs were always available (see Table 4).

**Table 4:** Drugs from the scheme

Quality of drugs	Frequency	Per cent
Yes	231	78.0
No	33	11.1
Don’t know	32	10.8
Total	296	100.0
**Were drugs prescribed under the scheme effective?**		
Yes	248	83.8
No	35	11.8
Don’t know	13	4.4
Total	296	100.0
**Availability of drugs issued under the scheme**		
Yes	135	45.6
No	141	47.6
Don’t know	20	6.8
Total	296	100.0

Staff availability for NHIS facilities was ranked low because, in facilities surveyed, staff were not readily available to attend to clients. Results showed that 65.5% indicated that they had to wait long before receiving treatment (see Table 5). Regarding difficulty in getting medicine, 54.4% never had difficulty getting medicine, while 42.6% encountered some difficulties getting medicine (see Table 5). Respondents were asked if locally made drugs were more available on the scheme than imported medicine, 49.3% didn’t know, 42.6% indicated “yes” and 8.1% indicated “no” (see Table 5).

**Table 5:** Staff availability

Do you wait long before receiving treatment?	Frequency	Per cent
Yes	194	65.5
No	93	31.4
Don’t know	9	3.0
Total	296	100.0
**Did you have difficulty getting medicine?**		
Yes	126	42.6
No	161	54.4
Don’t know	9	3.0
Total	296	100.0

### Qualitative data

#### Affordability of the scheme

Data collected from IDI respondents showed that the majority of respondents believed that the NHIS, which was designed to subsidize the cost of healthcare services for users, was causing private institutions to lose money; *the NHIS is structured in a way that the private institutions are at a loss as compared to the government institutions because the government institutions have subvention from the government, the government takes care of their overhead while the private work out what they use. So they are already skewed.”[SL IDI_1]. The price list of drugs presently is not anything to write home about because the NHIS price is very much lower than what is obtainable in the market. These prices are detrimental to the finance of the institution, in other words, if we continue, it is a way of running the hospital down, and the policy cannot survive for a long time because many private hospitals will opt out”[ETH IDI_2]. This is a real problem; NHIA is supposed to regulate all this and make sure private providers are not short-changed [ETH IDI_2]*.

#### Accessibility of the scheme

For the qualitative component, respondents (NHIS desk officers) did not feel there were discrepancies in access among socioeconomic groups. Although the majority of the respondents believed in the principle of free access to medications, a minority stated that access to medicines was limited due to the brand and type of medicines offered by pharmaceutical providers. Quotes from respondents showed that access had increased due to NHIS: *“NHIS has given room for people that cannot afford health care to have access to health care. It has actually tried to improve access. At least it has increased it a little bit. Some people usually just take anything from the patent medicine vendors but now they have access to [quality] medication… It has improved access.”[SL_IDI_].* Some of the IDI respondents also commented as follows “*…The scheme has improved the accessibility for the enrollees.”. [ETH_IDI_2]. You know many people are yet to understand the scheme but for the little, I have worked here the scheme is ok.” [SL_IDI_1].*

#### Availability of the medicines on the scheme

This is equally supported by findings from the IDI: “*The medicines provided are good ones” [AMH IDI_3]. “They give quality medicine. They give the best within the allocated funding,” Though I think more funding is required to increase the availability of drugs [RC IDI_4].* An IDI respondent opined that the availability of drugs under the NHIS scheme depended on the healthcare provider’s ability to make it accessible to the client *“It depends on the healthcare provider. I can rate it between 50 and 60% depending on the provider, but it’s individual access” [SL IDI_1]. I also think the government needs to step in to increase the allocation to medicine under NHIS, it will help [SL IDI _1]*.

On staff availability, most of the IDI respondents stated that many of the staff in NHIS-accredited facilities were not educated on what is expected or required of them concerning the scheme. They have very little knowledge of how to run the scheme. This is evidenced by the statement below: “*The desk officers are not even trained. The healthcare providers don’t even know what the scheme is all about… When you go to a hospital that is under NHIS, most times the staff don’t know what the scheme is all about”.(MoC_IDI_5)*.

## Discussion

Our study sought to assess the contextual nature of NHIS/FSSHIP, emphasizing accessibility, affordability, and availability (3As) and how focusing on these three can make NHIS a more beneficial and long-term scheme towards improving access to medicine. The results of our study have shown that focusing on these three can be achieved. The respondents reported that the NHIS improved their access to care regarding availability, affordability, and accessibility. Specifically, the NHIS was found to have increased the availability of essential drugs in accredited facilities, making it easier for enrollees to access the medications they needed. The scheme also played a significant role in reducing the financial burden of healthcare expenses, making healthcare more affordable for many Nigerians. Additionally, the NHIS improved the accessibility of healthcare services by ensuring that enrolled individuals had equitable access to healthcare facilities, regardless of their geographic location or socioeconomic status. These findings suggest that the NHIS has made significant strides in enhancing access to medicines in Nigeria, but there are still challenges that need to be addressed to ensure sustained access for all Nigerians.

### Accessibility

So far, the presence of the scheme alone has been reported to increase accessibility. It has made it possible for people to seek care from the hospital. This finding is consistent with other studies [17, 28] where NHIS increased accessibility and utilisation. However, this marginal increase in access to medicines under the NHIS is not enough as most of the country’s population is unemployed and not fully captured under the NHIS. The organisation and structure of Nigeria’s healthcare system appear to lack some of the essential components that could improve access to healthcare [29–33]. Obuaku [18] pointed out that there is inadequate access to healthcare services among a large percentage of the population and that, despite the reforms that have been made by the government, the majority of public health facilities are still short-staffed, ill-equipped, and low on medicine, vaccines, and treatment services. The implication of this for the sustainability of the scheme is that no matter the successes recorded so far by the scheme, a lot more would still need to be done, especially in the area of accessibility.

### Affordability

The respondents from our study indicated that NHIS improved service utilization. However, this improvement in utilisation tilts a great deal towards salaried workers of all cadres, most especially junior cadre workers. Through the scheme, low-income workers who would have otherwise not been able to afford essential medical services can now utilise such services without recourse to personal funds. However, the percentage that falls under this group (salaried workers) is minimal compared to the vast majority of unemployed citizens living across the country who are not able to utilise or have access to essential medical services. A lot more needs to be done to further improve access to medicine through the scheme’s affordability plans. The NHIS was designed to cover part of the costs (10%) of services. Due to the rising cost of health care in the country, the NHIS sought primarily to create a means through which health care could be affordable for all [17]. Thus, user fees are affordable for enrollees. The 10% co-payment paid by enrollees is the individual’s commitment to the scheme. This creates opportunities for low-income earners to afford health care. However, since the (NHIS/FSSHIP) scheme only covers salaried workers, the unemployed and those without any means of livelihood are still left to cater for the full cost of medical services unless they have a family member who enrolls them under their package. Despite how long the scheme has existed, it’s yet to go beyond the formal sector to cover those at the community level. Thus, total access to essential medicines is still beyond the reach of many.

### Availability

Availability was another subject raised during the course of this study. Availability of drugs and hospital staff are key factors in ensuring utilisation and access to medicine [34–36]. Our study findings showed that the drugs provided by the scheme, though of good quality, were not always available. Often, patients/clients were sent out to the drug stores outside the facility to buy needed drugs. This created some difficulties for patients who could not afford to buy medicines from private pharmacies, thus creating a barrier to access for those in need. This challenge (according to an IDI respondent) can be taken care of by using local pharmacies to dispense drugs free of charge, using the voucher issued to the patient by the doctor. This local pharmacy can then be reimbursed by the authorities [37]. In addition to the out-of-stock medicines, health facility staff were often not available. This meant that enrollees had to endure long waiting times before receiving treatment. Our study findings also showed that staff in many of the health facilities lacked adequate training needed in the performance of their duties, and were often clueless. This affected their relationship with clients and, by extension, access to essential medicine [18]. The country faces a shortage of healthcare institutions with adequate medical resources and staff to carry out the Scheme in an efficient manner [38].

### Challenges

The challenges with NHIS using the lens of the 3As of access revolved mostly around access to drugs and the availability of drugs, especially in private hospitals. The low price of medicines and low service charges insisted upon by the NHIS was considered a problem by some of the private hospitals, as they were barely able to meet their cost price and make a profit. If this continues, many hospitals might opt out of the NHIS, or provide separate services for respondents with better insurance service tariffs. In addition, although survey respondents indicated that the drugs prescribed under the scheme were reported as being of good quality and effective, some IDI respondents stated that They mentioned that *there are some adulterations; some people have adulterated some of these common drugs. When these clients take it, they don’t have the desired effect”*. Because of this, patients tend to “*lose confidence in some of these orthodox medicines”.*

Unavailability can be a deterrent to the accessibility of quality health care. If adequate facilities, skilled staff, and (quality) drugs were available and accessible but not affordable, the health service might not be used. A great number of Nigeria’s population still lives in poverty without adequate access to basic services and could benefit from more inclusive development policies [39]. Thus, affordability in a nation like Nigeria may be the link that holds all three together and may well be a “golden parachute” towards universal coverage. If people find health services affordable, the number of those seeking healthcare will increase, thus creating a stronger need for health services to be made available and accessible. It can therefore be said that NHIS improved access marginally, however, poor funding of healthcare in Nigeria has been a major barrier to the quality of healthcare service delivery [40]. Thus, the high burden of the costs of healthcare is being borne by individuals and households, which ranks Nigeria as the country with the second-highest level of out-of-pocket spending on health in the world [40]. To ensure effective and efficient operations of the NHIS, a possible link between the implementation of NHIS and corruption should be explored because the money meant to boost the health sector most often ends up in private pockets, which then results in inadequate funding to execute the programme effectively [41–43]. The country’s high level of corruption, lack of transparency, and accountability has negatively impacted the NHIS, making quality, accessible, and affordable healthcare difficult to provide [44, 45]. It is estimated that healthcare systems lose around 10% of their spending due to fraud or abuse, making it a critical issue [46, 47]. NHIA [48] reported that healthcare fraud costs the taxpayer 15% of healthcare spending on average, highlighting the enormity of this problem.

So, the implementation of the NHIS has faced several challenges such as insufficient staffing, physical health facilities, administrative and logistical impediments [49]. In addition, the processing of registered beneficiary documents and contributions to the NHIS, Health Maintenance Organisations (HMOs), and Health Providers (HPs) has been delayed, making managing the Scheme difficult [38] It is also challenging to organise the informal sector for the Scheme, and private clinics and hospitals are starting to reject the plan as well [38]. Due to these issues, NHIS is now vulnerable to various forms of fraudulent activity, despite the NHIA’s efforts to improve oversight and control measures.

Using the 3 A’s, it is evident that the National Health Insurance Scheme in Nigeria still faces significant challenges in providing sustained access to medicines. These challenges need to be addressed in order to ensure the effectiveness and success of NHIA’s efforts to improve oversight and control measures. The NHIA’s efforts to improve oversight and control measures have been commendable, but they have not completely eradicated fraudulent activity etc. This highlights the need for further interventions and strategies to ensure sustained access to medicines through the National Health Insurance Scheme in Nigeria. Additionally, addressing these challenges will require collaboration between stakeholders, including healthcare providers, regulatory bodies, and the government, to develop comprehensive solutions that prioritize patient welfare and combat fraudulent practices effectively.

### Study limitations and strengths

The study only focused on the health insurance scheme provided by the government as it is related to access to medicine. It, however, did not look at the fourth dimension (acceptability) of access to medicine and other health insurance schemes that are already in existence. Considering the limitations of scope, we suggested that future research could explore the dimension of acceptability more comprehensively, offering a more holistic understanding of healthcare access dynamics, public awareness, inadequate monitoring and evaluation; possible political interference, and even global health crises,

In using chi-square, we only tested for the association between variables. In addition, qualitative data was collected mainly from health insurance desk officers/managers, the inclusion of pharmacists might have revealed a bit more information, and further research should include the pharmacists.

The mixed methodology used in this study contributed to the study’s methodological rigour and is considered a strength.

## Conclusion

The study findings revealed that although the NHIS has successfully expanded access to medicines, there remain several challenges to its effective implementation and sustainability. Additionally, the scheme’s coverage of essential medicines could be improved even more, leading to reduced access to needed drugs for many Nigerians. A focus on the 3As (accessibility, affordability, and availability) for the scheme means that all facility categories (private and public) and their interests (where necessary) must be considered in further planning of the scheme to ensure that things work out well. Finally, the 3As become a focus when the governing body of the scheme takes complete charge of NHIS units in hospitals (private or public) as separate departments or liaison units, having government-employed staff (doctors, nurses, labs, equipment, attendants, etc.) in all these units across all facilities. For the private facilities, these staff will be answerable to and paid for by the government rather than the facilities they operate. Also, these staff will be properly trained for the tasks they are employed to carry out.

### Article summary

#### Strengths and limitations

- The mixed methodology used in this study contributes to the methodological rigor of the study.
- The study participants were only from one state.
- The study only focused on NHIS provided by the government as it is related to access to medicine….
- NHIS desk officers were the focus of IDIs.

### Electronic supplementary material

Below is the link to the electronic supplementary material.

Supplementary Material 1

Supplementary Material 2

## Data Availability

Technical appendix, statistical code, and dataset available from the lead author and the corresponding author.
