# Supplementary material for: Is the national health insurance scheme a pathway to sustained access to medicines in Nigeria?
Source: BMC Health Serv Res. 2024 Mar 29;24:403. doi: 10.1186/s12913-024-10827-1 (PMC10981341; doi:10.1186/s12913-024-10827-1)
Supplement: Supplementary file 1 — Supplementary Material 1 [file 12913_2024_10827_MOESM1_ESM.docx]

**IDI GUIDE**

1. What health system factors do you think influence access to essential medicine within the scheme?

**Probe for,** positive and negative influence, other factors

1. What benefit packages do you think will enhance access to essential medicine within the scheme?
2. In your own understanding, how would you rate access to medicine within FSSHIP?

**Probe for,** access outside the scheme

1. In your opinion, what category of individuals has more access within the scheme?

**Probe for,** low-income, middle-income and high income earners, response

1. Do you have any staff employed under scheme?
2. How are they paid?
3. What incentive/payment mechanisms are available in the scheme?
4. Do you think these payment mechanisms are enough to enhance access to medicine within the scheme?

**Probe response**

1. In your own understanding, how can access to essential medicine be improved?

**Probe for,** alternative means of funding, public-private partnership

1. What health financing options are best in ensuring a stable increase in access to medicine?
2. What can you say about the level of access to essential medicine for common communicable and non-communicable diseases within the scheme?

**Probe response**

1. What has been the scheme’s influence towards access to medicine?

**Probe for,** positive and negative influence

1. What is your perception about the medicines provided under the scheme?

**Probe for,** availability, affordability, acceptability, services under the scheme

1. In your opinion, what do you think are the challenges/barriers to the scheme?

**Probe for,** inadequate financing and infrastructure, weak governance, insufficient risk pooling, management staff inefficiency, inadequate staff etc.

1. How do you think these challenges/barriers can be tackled?

**Probe for,** making the scheme mandatory, improving regulation, exploring alternative funding etc.

1. In your opinion, what strategies can be used to guarantee full access to medicine?

**Probe for,** Availability, Accessibility, Affordability and Acceptability etc.
