# Supplementary material for: Is the national health insurance scheme a pathway to sustained access to medicines in Nigeria?
Source: BMC Health Serv Res. 2024 Mar 29;24:403. doi: 10.1186/s12913-024-10827-1 (PMC10981341; doi:10.1186/s12913-024-10827-1)
Supplement: Supplementary file 2 — Supplementary Material 2 [file 12913_2024_10827_MOESM2_ESM.docx]

**Access to essential medicines for major communicable and non-communicable diseases in the Nigerian Formal Sector Social Health Insurance Programme of the National Health Insurance Scheme (NHIS)**

| 1 | Survey record number | [ ] | Reserved for data entry |
| --- | --- | --- | --- |
| 2 | Organization name | [ ] | Enter the name of the facility where interview was conducted |
| 3 | Facility type | Primary [ ]  Secondary [ ]  Tertiary [ ]  Private [ ] |  |
| 4 | Interviewer initials |  |  |
| 5 | Date of interview |  | DD/MM/YYYY  //2017 |
| 6 | Respondent | [ ] | Primary beneficiary of FSSHIP Yes = 1; No = 0 |
| 7 | How many person are in your household | [ ] | *Please enter number only* |
| 8 | How many of your HH members are covered by health insurance (FSSHIP)? |  | *Please enter the first names or initials only* |
| 9 | Has any member of your household been ill in the past 6 months? | [ ] | Yes = 1; No = 0  *If ‘No’ end the interview and thank respondent* |

*(All of the following information – will be collected for each person who has been ill in the respondent’s household for the past 6 months)*

**PART ONE: DEMOGRAPHIC INFORMATION OF PERSON FOR WHOM INFORMATION IS COLLECTED**

|  |  |  |  |
| --- | --- | --- | --- |
| 10 | Name or initials of person for whom information is collected |  |  |
| 11 | Gender | [ ] | Male = 1; Female = 2 |
| 12 | Age in completed years |  |  |
| 13 | Age in completed months for infants < 1 year |  |  |
| 14 | Are you the primary beneficiary of FSSHIP | Yes [ ]  No [ ] | **(Remove)** |
| 15 | Relationship to FSSHIP primary beneficiary | [ ] | 1 = Spouse  2 = Child  3 = Ward  4 = Parent  8 = Other  9 = Not applicable |
| 16 | Marital status | [ ] | 1= Currently married  2 = Previously married  3 = Never married  9 = Not applicable |
| **Questions 17 and 18 are directed at Head of household if not primary beneficiary of FSSHIP** | | | |
| 17 | Highest level of education completed | [ ] | 0 = None  1 = Primary  2 = Secondary  3 = Tertiary |
| 18 | Employment status | [ ] | 1 = Unemployed  2 = Self-employed  3 = Paid employment  9 = Not applicable |

**PART TWO: ILLNESSES AND ACCESS TO HEALTH SERVICES**

| 19 | What kind of illness did the person for whom information is collected have in the past 6 months? | [ ] | 1 = Acute illness  2 = Chronic illness |
| --- | --- | --- | --- |
| 20 | What type of health problems did s/he have during the illness? |  | Specify the exact illness |
| 21 | When did the illness start? |  |  |
| 22 | How long have you had this illness? |  |  |
| 23 | At any point , did s/he (or anybody else on his/her behalf) seek care for this illness | [ ] | Yes = 1; No = 2 |
| 24 | From which of the following sources of care did s/he seek care for this illness outside the home? | Public hospitals/health centre [ ]  NGO/mission hospitals [ ]  Private health facilities [ ]  Private pharmacy [ ]  Patent medicine store [ ] | Yes = 1; No = 2 |
| 25 | How much time does it take to reach the following health care facilities that are closest to your household? | Public hospitals/health centre [ : ]  NGO/mission hospitals [ : ]  Private health facilities [ : ]  Private pharmacy [ : ]  Patent medicine store [ : ] | *Enter [HH:MM]*  *e.g.*  [01:30]  [00:30] |
| 26 | Did s/he take any medicine during the illness? | [ ] | Yes = 1; No = 2 |
| 27 | Which medicines were taken during this illness? |  |  |
| 28 | How much were you supposed to pay if you were not a beneficiary? | \|_\|\|_\|_\|_\|_\|_\|_\|_\| | (Naira) TOTAL COST |
| 29 | Was this cost covered by health insurance? | [ ] | Yes entirely = 1  Partly covered = 2  No = 3 |
| 30 | How much cost was covered by health insurance | [ ] (Naira) | If answer to Q 26 is partly covered |
| 31 | Did s/he take all medicines that were recommended or prescribed? | [ ] | Yes = 1; No = 2 |
| 32 | If No, what were the reasons? |  |  |
| 33 | Do you have any medicines available in your house? | [ ] | Yes = 1; No = 2 |
|  |  |  |  |

For each medicine listed, record the following information:

**PART THREE: OPINIONS ABOUT OBTAINING MEDICINES**

| 34 | Did the scheme improve your access to care | [ ] | Yes = 1  No = 2  I don’t know = 3 |
| --- | --- | --- | --- |
| 35 | In public facilities, do health providers take into account your ability to pay when they decide which medicines to prescribe? | [ ] | Yes = 1  No = 2  I don’t know = 3 |
| 36 | Are services (such as preventive health services amongst others) always available to you? | [ ] | Yes = 1  No = 2  I don’t know = 3 |
| 37 | Do you know if someone was ever exempted, regardless of insurance coverage? | [ ] | Yes = 1  No = 2  I don’t know = 3 |
| 38 | When you receive a prescription, are you comfortable that your insurance will cover it? | [ ] | Yes = 1  No = 2  I don’t know = 3 |
| 39 | When a pharmacist recommends a medicine, can you be sure that it is of good quality. | [ ] | Yes = 1  No = 2  I don’t know = 3 |
| 40 | Are all the medicines you purchase covered by the scheme? | [ ] | Yes = 1  No = 2  I don’t know = 3 |
| 41 | Are medicines for treatment of common medical condition, affordable for people with low – income? | [ ] | Yes = 1  No = 2  I don’t know = 3 |
| 42 | Medicines on the scheme are of good quality | [ ] | Yes = 1  No = 2  I don’t know = 3 |
| 43 | Are the medicines issued for NHIS in public facilities always available? | [ ] | Yes = 1  No = 2  I don’t know = 3 |
| 44 | Different names may be used for the same medicine | [ ] | Yes = 1  No = 2  I don’t know = 3 |
| 45 | Were the medicines effective? | [ ] | Yes = 1  No = 2  I don’t know = 3 |
| 46 | Did s/he have to wait long before receiving treatment? |  | Yes = 1  No = 2  I don’t know = 3 |

**if the medicines they had were made available by the scheme.

**PART FOUR: EXPERIENCES ABOUT MEDICINES**

| 47 | Medicines are more expensive at private pharmacies than at public health care facilities. | [ ] | Yes = 1  No = 2  I don’t know = 3 |
| --- | --- | --- | --- |
| 48 | Have s/he ever had difficulty getting medicine? | [ ] | Yes = 1  No = 2  I don’t know = 3 |
| 49 | Would your household obtain prescribed medicines if insurance reimbursed part of the cost | [ ] | Yes = 1  No = 2  I don’t know = 3 |
| 50 | Was the medicine correctly labelled with dose and expiry date | [ ] | Yes = 1  No = 2  I don’t know = 3 |
| 51 | Would you say the medicines you received are good quality | [ ] | Yes = 1  No = 2  I don’t know = 3 |
| 52 | locally manufactured medicines are more available on the scheme than Imported medicines | [ ] | Yes = 1  No = 2  I don’t know = 3 |

**PART FIVE: ASSETS AND MEDICINES EXPENDITURE**

| 53 | Can you please tell me how many rooms are in your home | \|_\|\|_\| | Include bedrooms, dining room, kitchen etc |
| --- | --- | --- | --- |
| 54 | Does your household own | Radio [ ]  Television [ ]  Generator [ ]  Bicycle [ ]  Motorcycle [ ]  Car [ ] | Yes = 1; No = 0 |
| 55 | Does your family have | House [ ]  Land/farmland [ ]  Livestock [ ] | Yes = 1; No = 0 |
| 56 | In the last week how much did your household spend on food | [ : ] Naira | Include the value of any food produced and consumed by the household; exclude alcohol, tobacco, and restaurant meals: |
| 57 | In the last week how much did your household spend on medicines | [ : ] Naira | Include cost of all medicines including orthodox and herbal, vitamins |
| 58 | Did your household spend on voluntary health insurance premiums or other prepaid plans? | [ ] | Yes = 1; No = 0 |
| 59 | In the last month how much did your household spend on Voluntary health insurance premiums or other prepaid health plans | [ : ] Naira |  |
| 60 | How much do you earn in a month (HH head) | [ ] |  |
| 61 | Is there another major source of income? | Yes [ ]  No [ ] | Yes = 1; No = 0 |
| 62 | If yes please specify | [ ] |  |
